# Modern Therapeutics: A Compendium of Recent Formulæ and Specific Therapeutical Directions

**Published:** 1871-05

**Authors:** 


Modern Therapeutics: a Compendium of Recent Formulæ and Specific Therapeutical Directions. By George H. Napheys, A.M., M.D. Second edition, revised and improved. Published by S. W. Butler, M.D., 115 S. Seventh St., Philadelphia; for sale by W. B. Keen & Cooke, Chicago.

This is a volume of 400 pages, published in good style, and neat cloth binding.

It contains, in addition to "Recent Formulæ," "Specific Therapeutical Directions," and, to some extent, the philosophy thereof, in the management of disease.

It also differs from other collections of therapeutical facts, in that it is arranged with reference to the diseases, instead of according to the articles of the materia medica, thus enabling one to turn at once to the therapeusis of any particular disease.

